# Elements of Surgery

**Published:** 1846-03

**Authors:** 


Art. X.—*Elements of Surgery.* By Robert Liston, Surgeon to the North London Hospital, Professor of Clinical Surgery, &c. &c. &c. Fourth American from the last London edition; *with copious Notes* and Additions; by Samuel D. Gross, M. D., Professor of Surgery in the Medical Institute of Louisville, Surgeon to the Louisville Marine Hospital, &c. &c. Illustrated with numerous engravings. Philadelphia: Ed. Barrington & Geo. D. Haswell; Louisville, Ky.: James Maxwell. 1846. (8vo. pp. 664.)

Few works on Surgery have been more popular than the Elements of Mr. Liston. A large edition was issued three years ago, and the publishers now offer to the medical public a fourth, enriched by the indefatigable editor with the modern discoveries in surgical science. The author well remarks in his preface, that "the rapid advancement made in the pathology and treatment of surgical diseases demands a perpetual revision and correction of the systematic works devoted to this department of the healing art." In this a mutual obligation is implied between authors and practitioners. As it is the duty of the first to reflect the existing state of the science, keeping pace with discovery, so it is the duty of the latter to inform themselves of all that is added, from time to time, to the curative resources of the profession. The authors have faithfully performed their duty in the execution of this work; and its rapid sale shows that practitioners are not unmindful of theirs.

